# Maturation of Elasticity, Pliability and Colour of Thin Split‐Thickness Skin Grafts on Deep Dermal and Muscular Wound Bed Within the Course of 1 Year

**DOI:** 10.1111/iwj.70944

**Published:** 2026-05-14

**Authors:** Claudius Illg, Alexa Gaag, Katarzyna Rachunek‐Medved, Johannes Tobias Thiel, Adrien Daigeler, Sabrina Krauss

**Affiliations:** ^1^ Department of Hand, Plastic, Reconstructive and Burn Surgery, BG Unfallklinik Tuebingen Eberhard Karls University Tuebingen Germany

**Keywords:** Cutometer, Mexameter, scar scale, skin elasticity, split‐thickness skin graft

## Abstract

Split‐thickness skin grafts are commonly used for reconstruction of soft tissue defects with a well‐vascularised wound bed. This study evaluated changes in scar elasticity, pliability, erythema and pigmentation during scar maturation and compared scars on deep dermal versus muscular wound beds. Seventeen graft scars on deep dermal wound beds and five on muscular wound beds were assessed at 1, 3, 6 and 12 months postoperatively using the Vancouver, Hamilton and Manchester scar scales, as well as a visual analog scale. Objective measurements included skin elasticity and pliability (Cutometer) and erythema and melanin content (Mexameter). All scar scale scores improved significantly over 12 months. Skin pliability increased continuously and remained higher in deep dermal wound beds than in muscular wound beds. Skin elasticity was initially slightly increased compared to healthy skin. The erythema index decreased over time, while the melanin index increased, both approaching control values. Melanin levels were consistently higher in deep dermal wound beds and increased more rapidly at scar margins. Overall, split‐thickness skin graft scars showed prolonged maturation, with only elasticity and melanin content reaching healthy skin levels within 12 months. Preservation of the deep dermis, when possible, may improve scar quality.

## Key Points

- Split‐thickness skin graft scar maturation is a prolonged process that continues for at least 12 months.
- Skin elasticity is initially slightly increased compared with healthy skin and reaches levels comparable to healthy skin within 12 months.
- Skin pliability increases continuously, approaching but not reaching normal values.
- Erythema continuously decreases while melanin content increases; however, only melanin content returns to levels comparable to those of healthy skin within 12 months, with a faster increase at the scar margins.
- The scar quality of split‐thickness skin grafts can be improved, with increased scar pliability and melanin content, by preserving the deep dermis as the wound bed, which the authors recommend whenever possible.

## 1 Introduction

In large soft tissue defects, split‐thickness skin grafts (STSGs) are a good option for wound closure when a well‐vascularised and clean wound bed, such as the deep dermis or muscle, is given. Meshing of STSGs can be used to expand the transplants and thereby allow the coverage of a larger recipient area and also provide fluid and blood drainage [1, 2]. The STSGs are initially supplied by imbibition, which is why thin STSGs have an advantage in nutrition compared to thick STSGs [3]. Inosculation and revascularisation of the graft follow until postoperative Days 5–7 [4]. In the case of meshed STSGs, the epithelialisation process in the gaps within the transplant originates from the margins of the skin bridges [5]. The maturation of the skin graft then follows, including changes in raising, pigmentation, redness, rigidity and elasticity [6, 7, 8].

Besides the closure of the skin defect and consequently the restitution of the barrier function, the temperature regulation and avoidance of excessive fluid loss, another important goal of the skin graft is an aesthetically pleasing outcome with a smooth and flexible surface. Not least because large scars in particular are often visible in everyday life. To achieve this, texture, pigmentation and colour should match the recipient site in the long term.

Scar scales provide a useful tool that is easily applicable in daily clinical practice to record these factors and monitor scar maturation [9]. However, several different scar scales with distinct items and assessment criteria impede comparability [10]. Furthermore, the evaluation should ideally be conducted by the same objective assessor to ensure consistent results [11].

A more objective assessment of scar characteristics and quality can be achieved with specialised noninvasive tools [12]. Scar colour, in terms of erythema and melanin, can be assessed with spectrophotometric devices, while elasticity and pliability of the scar can be quantified by applying a defined amount of suction on the scar and measuring the tissue movement [13, 14].

The wound bed is usually given, whereby it should be considered that the type of wound bed does not influence the STSG take rate, provided that the wound bed perfusion is adequate [15]. However, it is unclear whether deep dermal wound beds and muscular wound beds provide comparable scar quality in the mid‐ and long‐term.

The aim of this study was to determine whether there is a difference in STSG scar quality and in the scar maturation process between deep dermal and muscular STSG scars and whether the centre and margins of the scars mature uniformly.

## 2 Methods

The present prospective study was conducted at our institution after approval by the local Research Ethics Committee (project number 289/2020BO2) in accordance with the Declaration of Helsinki. All competent patients of legal age who received STSGs on a deep dermal wound bed or on muscle/muscle fascia between August 2021 and December 2022 in our department were provided with oral and written information, and all consenting volunteers were included. Patients that received STSGs on free, pedicled or local flaps were not included. Patients with STSGs retaken at a donor site that was previously used as STSG donor site were excluded. Patients were also excluded if they had peripheral artery occlusive disease, liver dysfunction or were over 80 years of age. All participants gave written informed consent. Age, sex, comorbidities, smoking status and the location of the STSGs were noted.

### 2.1 STSGs Harvest and Transplantation

STSGs were harvested with an electric‐powered dermatome in 2/10 mm thickness. The STSGs were then meshed with a meshing device at a ratio of 1:1.5 and transplanted onto a vital and clean deep dermal or muscular/epifascial wound beds. The STSGs were secured with surgical staples and a tie‐over dressing consisting of one layer of paraffin gauze and a polyurethane sponge. The tie‐over dressing was removed after 5 days, followed by paraffin gauze dressings that were changed every 1–3 days until complete healing. Patients were encouraged to apply lipid ointment daily after wound healing and avoid sun exposure when possible, or otherwise apply sunscreen with SPF 50+ for 1 year after the transplantation, which is synonymous with the entire follow‐up period.

### 2.2 Examination

The STSG scars were examined and evaluated at 1, 3, 6 and 12 months after grafting at the exact same location during each follow‐up. Photographs were taken to document the measurement site to facilitate relocation for serial measurements. For each STSG scar, two areas were investigated, one in the middle of the scar and one 10 mm from the edge of the scar. Both values were compared, and the mean of these two values was calculated and used for the comparison of deep dermal and muscular transplants. Furthermore, a control measurement was conducted on healthy tissue at the mirrored location of the scar. On the examination day, no ointment was applied. During each follow‐up examination, scars and controls were exposed, and patients were placed in a comfortable position on an examination couch. All measurements were conducted in a standardised fashion. The STSG scars were evaluated with scar scales by the same independent examiner who was not involved in the patients' clinical treatment. Elasticity and pliability parameters were then measured with the Cutometer, and the melanin and haemoglobin indices were assessed with the Mexameter, as described in detail below.

### 2.3 Scar Scales

The same examiner evaluated each scar during each follow‐up using three established scar scales, including the Vancouver Scar Scale, the Hamilton Scar Scale and the Manchester Scar Scale. Furthermore, each patient rated their scars of interest on a visual analogue scale from 0 to 10, with 0 being the best and 10 being the worst outcome concerning the items pain, tension, itching and skin quality and a comprehensive subjective score was calculated by adding the single item scores.

### 2.4 Cutometer

To objectify mechanical skin properties, the Cutometer Dual MPA 580 (Courage + Khazaka electronic GmbH, Cologne, Germany), which utilises a laser to assess the speed and amount of skin displacement induced by a specific negative pressure, allows conclusions to be drawn on skin elasticity. A Cutometer probe with a 6 mm diameter opening was used in all investigated locations. Measurement Mode 1 (time‐strain‐mode) was selected with a 2 s suction phase with a constant suction of 450 mbar and a 2 s relaxation phase. Each measurement comprised a total of three sequential cycles. The parameters R0 (maximum amplitude at the end of the suction phase in mm, represents skin pliability), R2 (gross skin elasticity, representing the skin's ability to return to its original state during the relaxation phase), R3 (maximum amplitude at the end of the suction phase of the last suction curve in mm, represents tiring effects) and R5 (net elasticity, representing the ratio between the initial [elastic] part of the suction phase and the immediate recovery during relaxation) were analysed. These parameters were selected because the parameter R0 is an important measure of skin pliability and the parameters R2 and R5 are important measures of elasticity. Furthermore, R0 and R3 are characterised by a high interrater reliability and are well suited for comparisons of STSGs and normal skin [12, 16].

### 2.5 Mexameter

The Mexameter MX 18 (Courage + Khazaka electronic GmbH, Cologne, Germany) was applied to determine skin melanin content by measuring the spectral absorption rate at wavelengths of 660 and 880 nm, and haemoglobin content, representing erythema, was assessed at wavelengths of 568 and 660 nm. The device outputs relative score values between 0 and 999; higher melanin or haemoglobin content increases the values.

### 2.6 Statistics

For statistical analysis, ordinary one‐way analysis of variance was performed for multiple comparisons, while two‐tailed unpaired or paired *t* tests were performed for two‐sample comparisons with GraphPad Prism v. 9.5.0 (GraphPad Software, La Jolla, CA). Values are given as mean ± standard deviation, and the range is provided when appropriate. A *p* < 0.05 was considered statistically significant.

## 3 Results

Twenty‐three voluntary patients met the inclusion criteria. Four of these patients were excluded in retrospect because of incomplete measurement data or dropout during the follow‐up period. Nineteen patients with a mean age of 53.2 ± 17.2 years (range 21–79 years), including six females (31.6%), were included. In three patients, two scars on different areas were included, resulting in a total of 22 STSG scars. Seven patients were active smokers. The wound bed of the recipient site consisted of deep dermis in 17 scars and of muscle and muscle fascia in five scars. The soft tissue defect measured 1.2% ± 0.6% of total body surface area (range 0.25%–3.0%) in the deep dermal group and 3.9% ± 3.8% (range 0.5%–10.0%) in the muscle group. The scars were predominantly located on the extremities. More specifically, within the deep dermal group, seven scars (41%) were located on the upper extremity, eight (47%) on the lower extremity, one (6%) on the trunk and one (6%) on the head, whereas in the muscular group, one scar (20%) was located on the upper extremity, two (40%) on the lower extremity and two (40%) on the trunk. None of the scars covered more than 50% of the extremity or trunk circumference.

To investigate the time course of STSG maturation, the first part of the results section presents a combined analysis of the values for deep dermal and muscular scars. In the second part, the differences between deep dermal and muscular scars are assessed.

### 3.1 Combined Assessment of the Time Course of STSG Maturation in Deep Dermal and Muscular Scars

#### 3.1.1 Scar Scales

**FIGURE 1:**
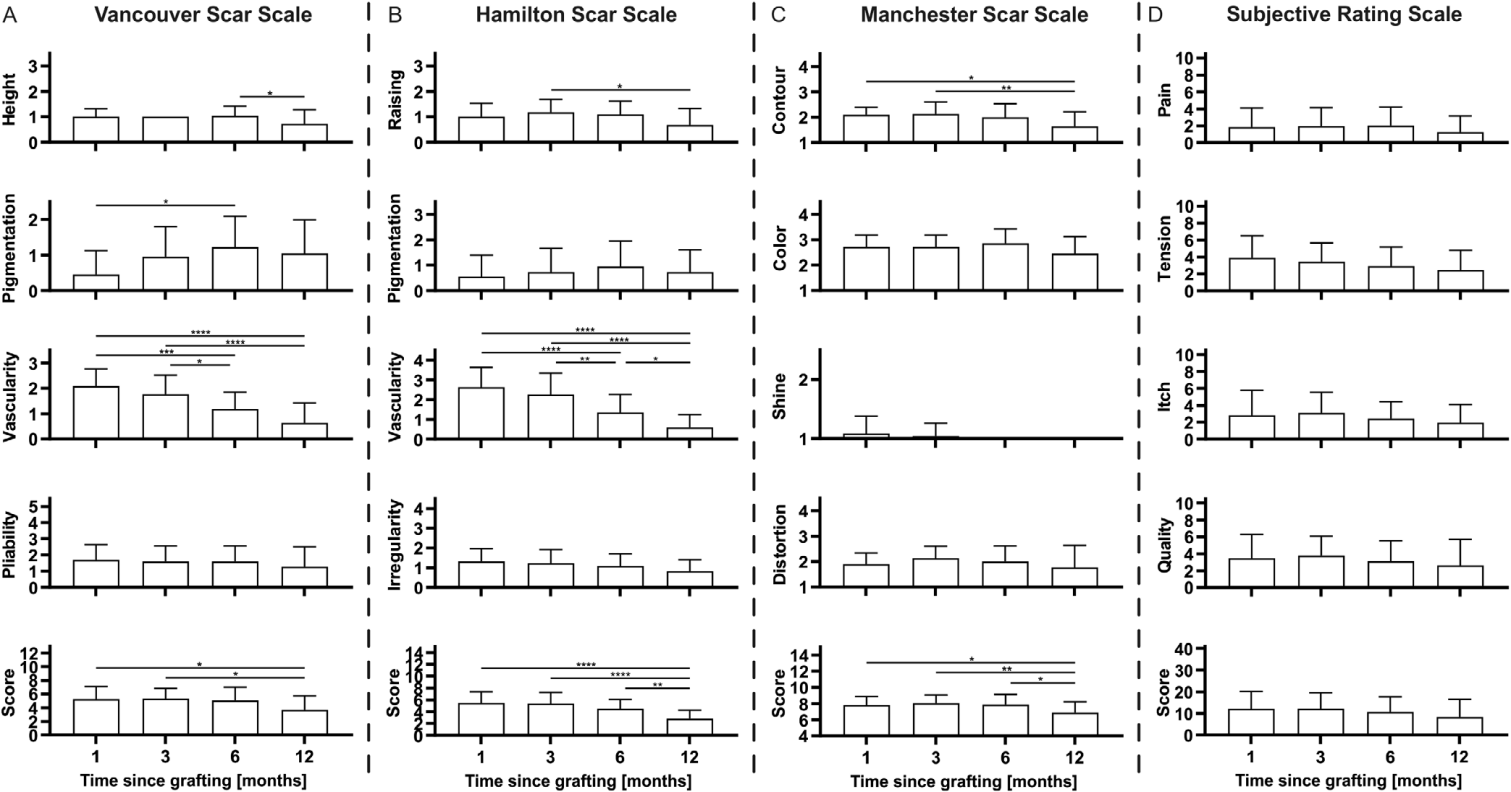
The development within 1 year of skin grafting of all separate items and the total score values of the Vancouver Scar Scale (A), the Hamilton Scar Scale (B), the Manchester Scar Scale (C) and the subjective visual rating scale (D) are visualised by bar graphs. The four scales are separated by dashed lines. *N* = 22, ordinary one‐way analysis of variance, *p < 0.05; **p < 0.01; ***p < 0.001; ****p < 0.0001.

Significant changes were observed in individual items on all three scar scales, as well as on the total scores of all three scales. The total scores improved (decreased) significantly on all scales comparing Months 1 and 12 (Vancouver Scar Scale: 5.23 ± 1.82 vs. 3.69 ± 2.06, *p* = 0.034; Hamilton Scar Scale: 5.50 ± 1.85 vs. 2.82 ± 1.44, *p* < 0.0001; Manchester Scar Scale: 2.09 ± 0.29 vs. 1.64 ± 0.58, *p* = 0.013), as well as Months 3 and 12. The Hamilton and Manchester scar scales furthermore improved comparing Months 6 and 12. The major improvements occurred therefore after 6 months postoperatively. The contour significantly improved within 12 months when assessed with the Manchester Scar Scale (2.09 ± 0.29 vs. 1.64 ± 0.58, *n* = 22, *p* = 0.13). Furthermore, height on the Vancouver Scar Scale improved significantly from Months 6 to 12 (1.05 ± 0.38 vs. 0.73 ± 0.55, *n* = 22, *p* = 0.03), and raising on the Hamilton Scar Scale improved significantly from Months 3 to 12 (1.18 ± 0.50 vs. 0.68 ± 0.65, *n* = 22, *p* = 0.019). The items pigmentation and colour as well as the items pliability, irregularity and distortion, were not subject to substantial changes within the 12‐month follow‐up period. The largest improvement was seen on the item vascularity on the Hamilton and Vancouver scar scales when comparing 1 and 12 months postoperatively, with a significant score decrease (2.64 ± 1.00 vs. 0.59 ± 0.67, *n* = 22, *p* < 0.0001 and 2.09 ± 0.68 vs. 0.64 ± 0.79, *n* = 22, *p* < 0.0001). However, on the subjective visual rating scale, all items did not undergo a significant change within the 12 months follow‐up period (Figure 1).

#### 3.1.2 Cutometer

**FIGURE 2:**
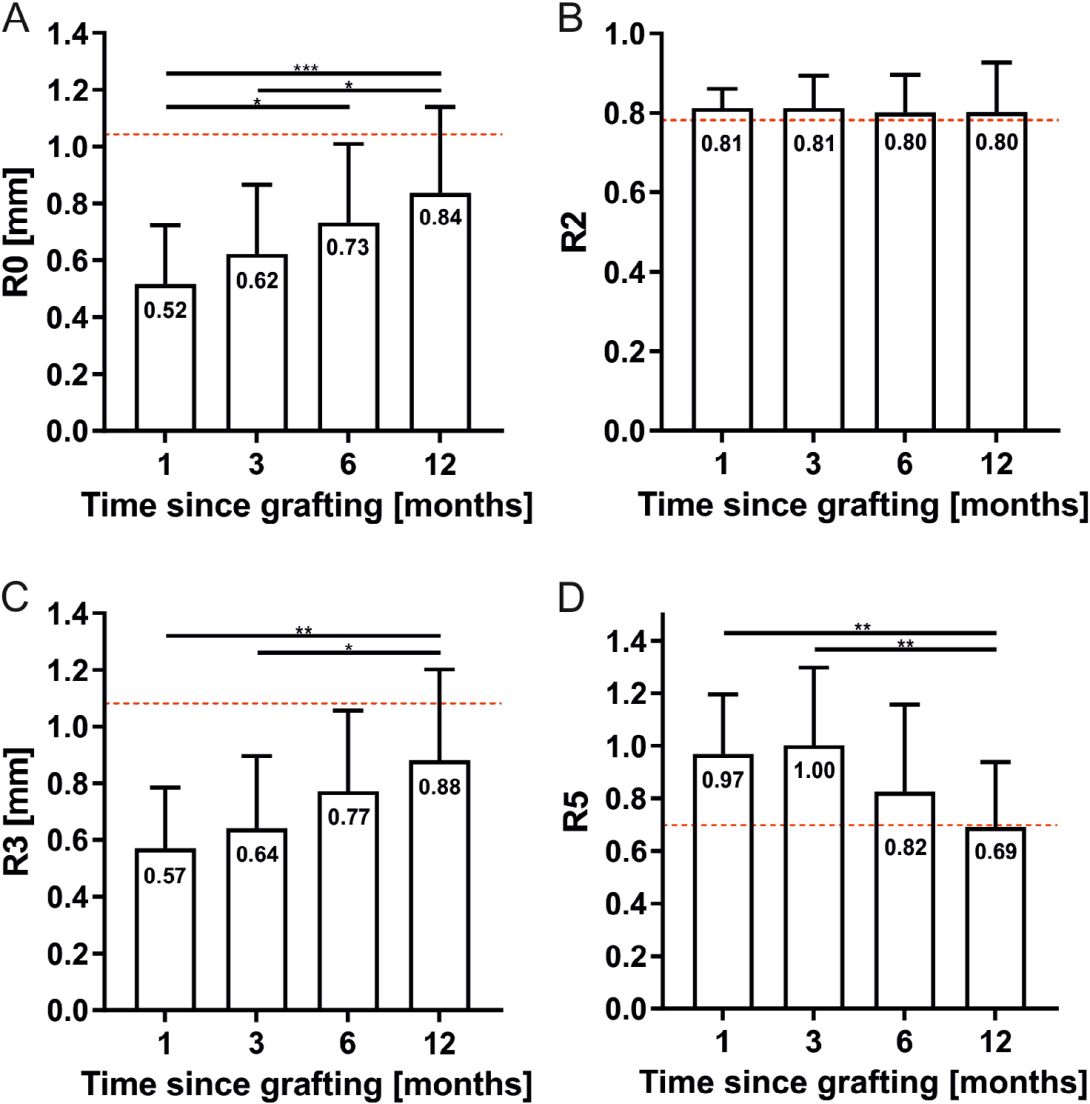
The Cutometer values R0 (A), R2 (B), R3 (C) and R5 (D) are illustrated with bar graphs for all four follow‐up times. The graphs show a gradual increase in skin pliability (A and C), while gross elasticity remains constant (B) and net elasticity decreases to finally reach the control value (D). The mean is given and the mean value of the control, representing the normative value, is indicated by the dotted red line. *N* = 22, ordinary one‐way analysis of variance, *p < 0.05; **p < 0.01; ***p < 0.001.

The parameter R0, representing the skin pliability or firmness, continuously increased significantly from 1 month postoperatively (0.52 ± 0.21 mm, *n* = 22) to 12 months postoperatively (0.84 ± 0.30 mm, *n* = 22; *p* = 0.0005). However, the parameter R0 did not reach the level of the control within the follow‐up period (12‐month control: 1.19 ± 0.31 mm, *n* = 22; *p* < 0.0001) (Figure 2A).

The parameter R2, representing the skin's ability to return to its original state during the relaxation phase, showed no significant dynamic within the 12‐month follow‐up period (1 month: 0.81 ± 0.048, *n* = 22, 12 months: 0.80 ± 0.13, *n* = 22, *p* = 0.98). Furthermore, there was no significant difference when compared to the healthy skin (mean: 0.81 ± 0.090, *n* = 88; control mean: 0.78 ± 0.12, *n* = 22, 0.13) (Figure 2B).

The parameter R3, representing the maximum amplitude at the end of the suction phase of the last suction curve in mm, increased continuously in concordance with R0 from 1 month postoperatively (0.57 ± 0.22 mm, *n* = 22) to 12 months postoperatively (0.88 ± 0.32 mm, *n* = 22; *p* = 0.0015) (Figure 2C). The difference between R0 and R3 (equivalent to parameter R9) was constant within the follow‐up period (*p* > 0.14) with a mean of 0.037 ± 0.041 mm.

The parameter R5, representing the ratio between the initial (elastic) part of the suction phase and the immediate recovery during relaxation, decreased within 12 months (1 month: 0.97 ± 0.23, *n* = 22; 12 months: 0.69 ± 0.25, *n* = 22, *p* = 0.0073) and reached the level of the control 12 months postoperatively (0.60 ± 0.23, *n* = 22, *p* = 0.11) (Figure 2D).

The centre and margin values of the four parameters showed a significant difference only for the parameter R0 at 3 months postoperatively (centre: 0.53 ± 0.24, *n* = 22; margin: 0.71 ± 0.37, *n* = 22, *p* = 0.048).

#### 3.1.3 Mexameter

**FIGURE 3:**
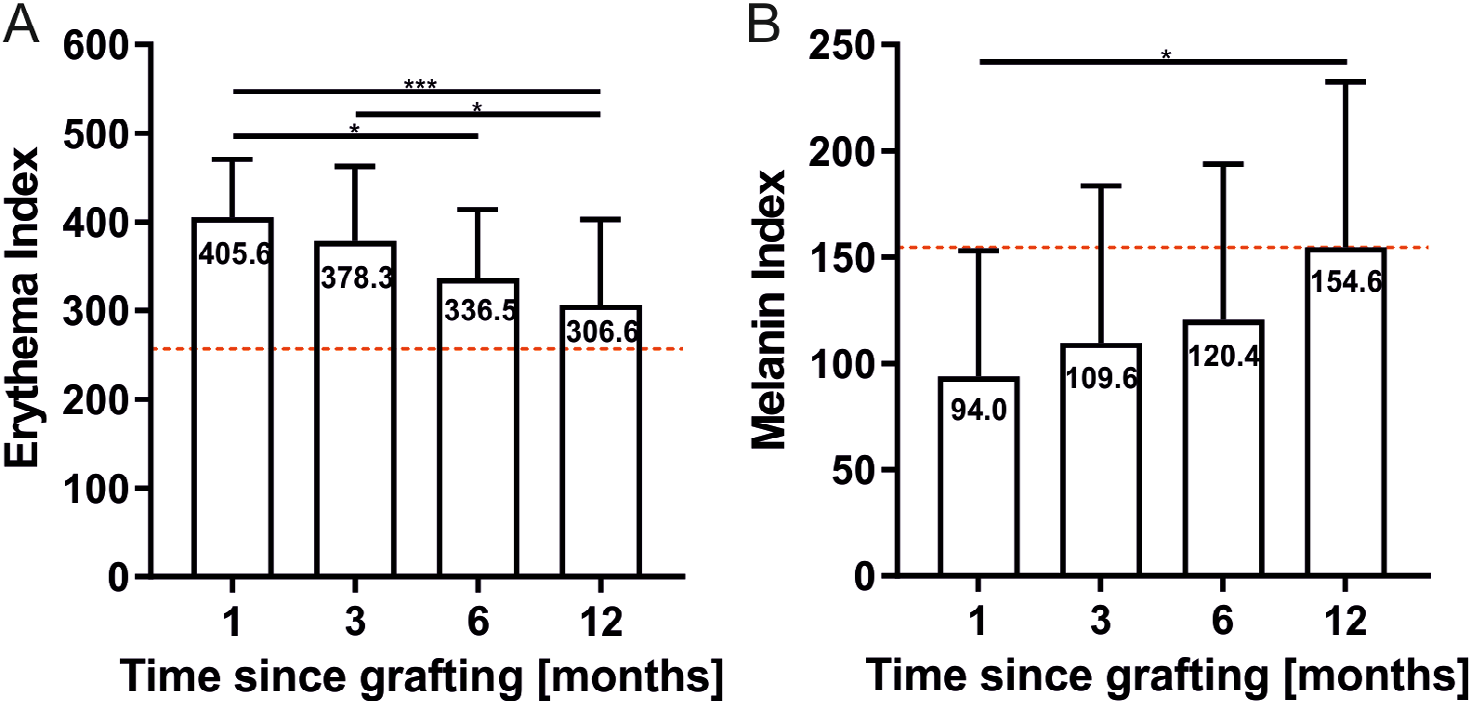
The erythema index is illustrated with bar graphs (A), and a significant decrease of the erythema over time becomes apparent. The melanin index is illustrated with bar graphs (B) and shows a significant increase in melanin within the 12‐month follow‐up period. The mean is given. The dotted red line corresponds to the mean control value, representing the norm value. *N* = 22, ordinary one‐way analysis of variance, *p < 0.05; ***p < 0.001.

A continuous decrease of the erythema index was apparent over time, corresponding with a convergence of the erythema index of the scar toward the control values in the course of time (1 month: 405.6 ± 65.4, *n* = 22; 12 months: 306.6 ± 65.4, *n* = 22, *p* = 0.0007). However, the baseline was not reached until the end of the 12‐month follow‐up period (12‐month control: 257.7 ± 102.9, *n* = 22; *p* = 0.11) (Figure 3A). The control values itself remained stable between each follow‐up. The melanin index values continuously increased during the observation period from an initial value of 94.0 ± 59.0 (*n* = 22) to 154.6 ± 77.7 (*n* = 22, *p* = 0.030) 12 months postoperatively, almost reaching the 12‐month control value (156.5 ± 70.8, *n* = 22, *p* = 0.93) (Figure 3B). The erythema and melanin index values were not significantly different in the centre and the margin of the scar (*p* > 0.23).

### 3.2 Comparison of STSG Maturation in Deep Dermal and Muscular Scars

#### 3.2.1 Comparison of Deep Dermal and Muscular STSGs—Scar Scales

**FIGURE 4:**
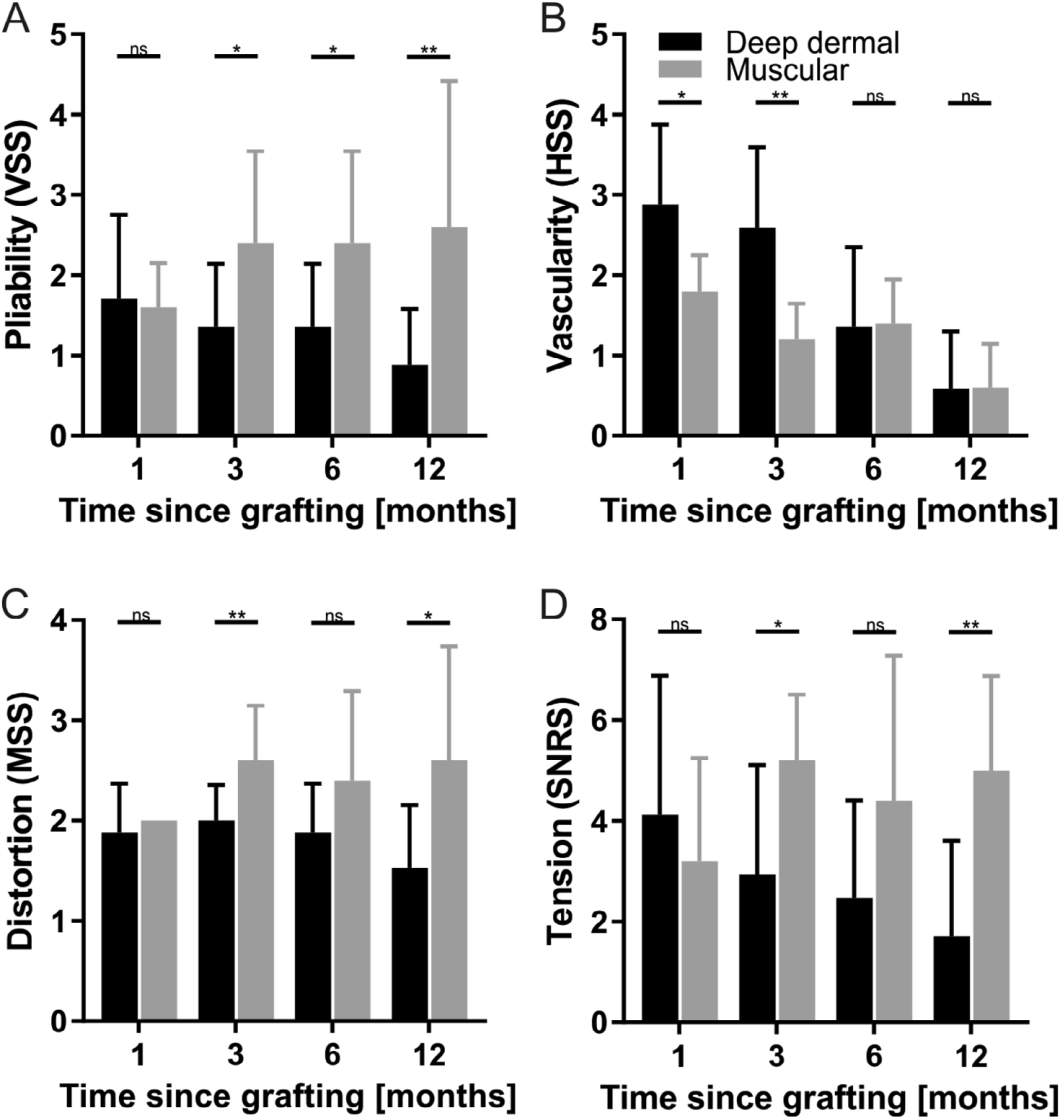
Singular scar scale items with significant differences between STSGs on deep dermal (black) and muscular (grey) wound bed within the follow‐up period are illustrated with bar graphs. In (A), the countervailing trend of pliability measured with the Vancouver Scar Scale (VSS) becomes apparent. The vascularity measured with the Hamilton Scar Scale (HSS) reveals a synchronous trend of STSGs on deep dermal and muscular wound bed, with initially increased vascularity in deep dermal STSGs (B). The distortion, assessed with the Manchester Scar Scale (MSS) and the tension, assessed with the subjective visual rating scale (SVRS) show opposing trends (C and D). *N* = 5 for deep dermal wound bed and *N* = 17 for muscular wound bed, two‐tailed unpaired *t* test, *p < 0.05; **p < 0.01; ns, not significant.

The comparison of the scar scales' score values did not show significant differences comparing STSGs on deep dermal and muscular wound bed at the beginning or the end of the investigation period. However, several individual items showed significant differences. While the pliability, rated by the Vancouver Scar Scale, was comparable in the deep dermal and muscular groups 1 month after operation (1.71 ± 1.05, *n* = 17 vs. 1.60 ± 0.55, *n* = 5; *p* = 0.83), the pliability score continuously decreased in deep dermal transplants, meaning that the tissue became more pliable and increased in muscular transplants within the measurement period, resulting in a significant difference 12 months after transplantation (0.88 ± 0.70, *n* = 17 vs. 2.60 ± 1.82, *n* = 5; *p* = 0.036) (Figure 4A). Furthermore, the Hamilton Scar Scale showed an increased vascularity in deep dermal STSGs when compared to muscular STSGs initially (2.88 ± 1.00, *n* = 17 vs. 1.80 ± 0.45, *n* = 5; *p* = 0.030) and an approximation of the vascularity in both groups 12 months after transplantation (0.59 ± 0.71, *n* = 17 vs. 0.60 ± 0.55, *n* = 5; *p* = 0.97) (Figure 4B). Moreover, the scar distortion, measured with the Manchester Scar Scale, was comparable in deep dermal and muscular STSGs 1 month postoperatively (1.88 ± 0.49, *n* = 17 vs. 2.00 ± 0.0, *n* = 5; *p* = 0.60), and showed a decrease in deep dermal STSGs and an increase in muscular STSGs during the measurement period, resulting in a significant difference after 12 months (1.53 ± 0.62, *n* = 17 vs. 2.60 ± 1.14, *n* = 5; *p* = 0.011) (Figure 4C). Furthermore, the subjective visual rating scale revealed a decreasing tension in deep dermal STSGs and an increasing tension in muscular STSGs (1 month: 4.12 ± 2.76, *n* = 17 vs. 3.20 ± 2.05, *n* = 5; *p* = 0.50; 12 months: 1.71 ± 1.90, *n* = 17 vs. 5.00 ± 1.87, *n* = 5; *p* = 0.027) (Figure 4D).

#### 3.2.2 Comparison of Deep Dermal and Muscular STSGs—Cutometer

**FIGURE 5:**
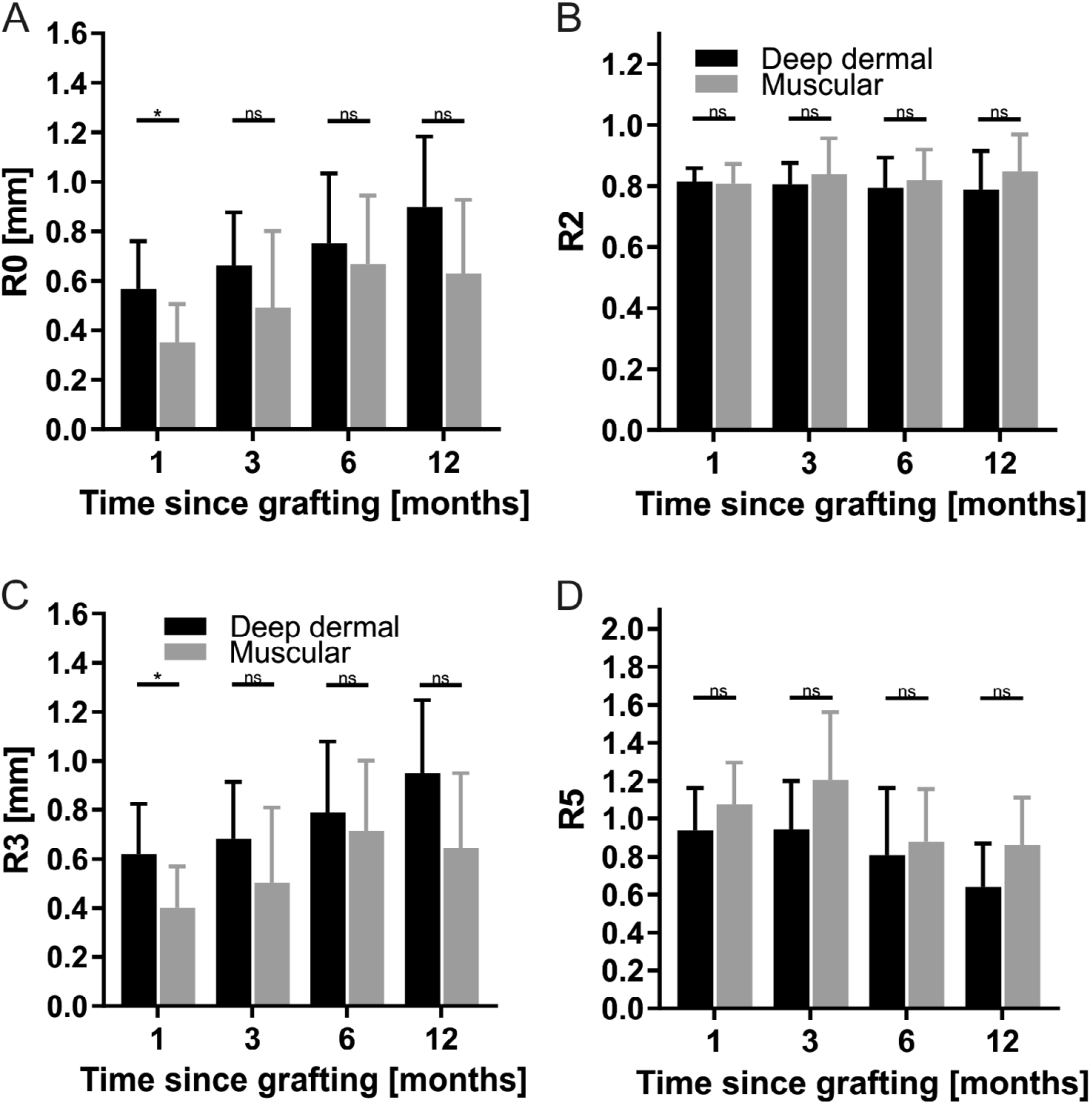
The Cutometer values R0 (A), R2 (B), R3 (C) and R5 (D) of STSGs on deep dermal (black) and muscular (grey) wound bed are visualised with grouped bar graphs for comparison at 1, 3, 6 and 12 months postoperatively. *N* = 5 for deep dermal wound bed and *N* = 17 for muscular wound bed, two‐tailed unpaired *t* test, *p < 0.05; ns, not significant.

The comparison of the Cutometer values R0, R2, R3 and R5 between deep dermal and muscular STSGs revealed a uniform dynamic in both groups. The parameters R0 and R2 were increased in deep dermal STSGs when compared to muscular STSGs throughout the entire period, with significance 1 month postoperatively (R0: 0.57 ± 0.20, *n* = 17 vs. 0.30 ± 0.16, *n* = 5, *p* = 0.036; R3: 0.62 ± 0.21, *n* = 17 vs. 0.40 ± 0.17, *n* = 5, *p* = 0.043). However, the parameter R2 did not show significant differences comparing STSGs on deep dermal and muscular wound beds (mean: 0.80 ± 0.088, *n* = 68 vs. 0.83 ± 0.097, *n* = 20, *p* = 0.24), while the parameter R5 was consistently elevated in STSGs on muscular wound beds, with statistical significance in the mean across all four follow‐up times (0.83 ± 0.29, *n* = 68 vs. 1.0 ± 0.30, *n* = 20; *p* = 0.022) (Figure 5).

#### 3.2.3 Comparison of Deep Dermal and Muscular STSGs—Mexameter

**FIGURE 6:**
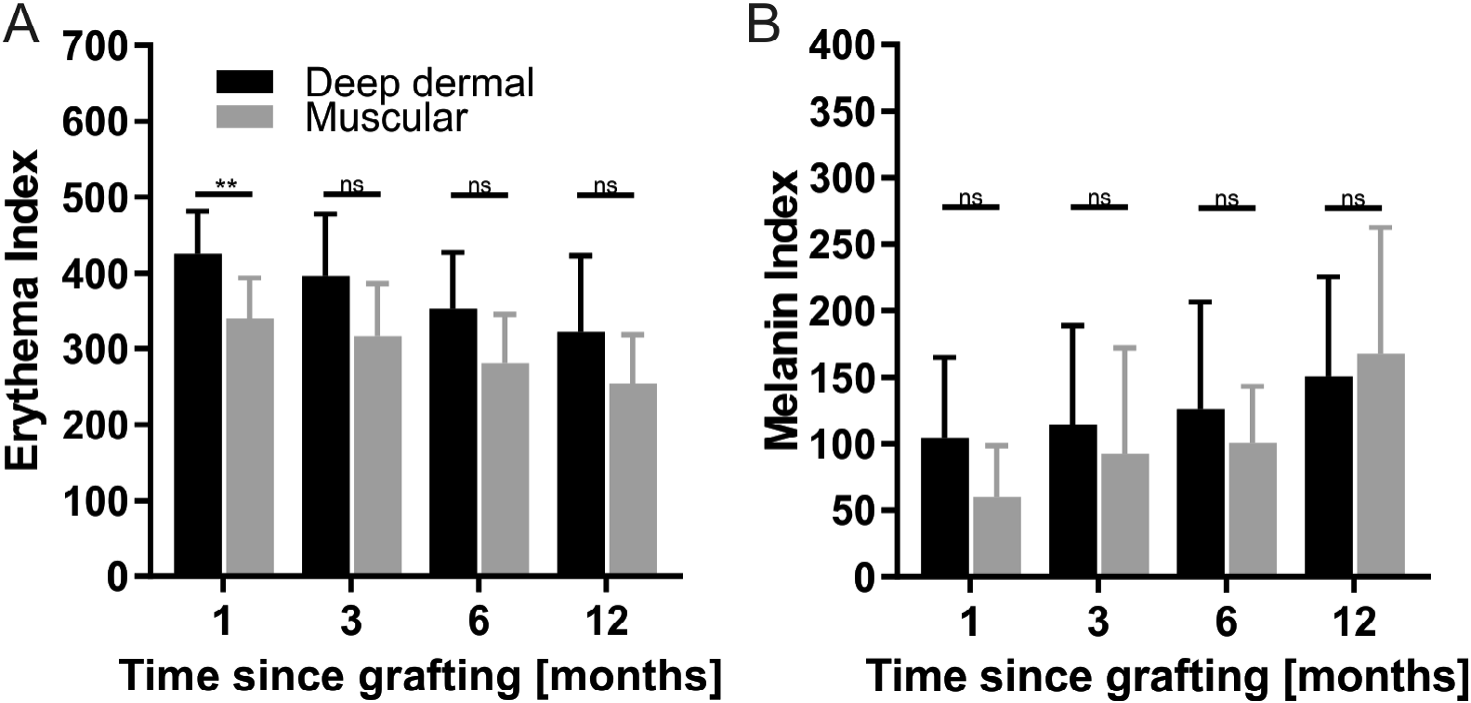
The erythema index (A) and the melanin index (B) are illustrated with bar graphs to compare deep dermal (black) and muscular (grey) STSGs, which show a uniform trend during the 12 month follow‐up period. *N* = 5 for deep dermal wound bed and *N* = 17 for muscular wound bed, two‐tailed unpaired *t* test, **p < 0.01, ns, not significant.

The comparison of the melanin index and the erythema index in deep dermal and muscular transplants showed a synchronous trend during the follow‐up period. The erythema index was constantly higher in the deep dermal group, with an overall mean of 374.0 ± 87.6 (*n* = 68) versus 298.1 ± 66.8 (*n* = 20), with statistical significance (*p* = 0.0006). Furthermore, the melanin index was higher in the deep dermal group initially (Month 1: 104.0 ± 61.0, *n* = 17 vs. 60.0 ± 38.7, *n* = 5, *p* = 0.15), but without statistical significance, and values were similar 12 months postoperatively (Figure 6).

## 4 Discussion

For soft tissue defects with a well‐vascularised wound bed, STSG are an essential tool in the plastic and reconstructive surgeon's toolbox. However, the scar can lead to contractures and can be aesthetically unpleasing. The aim of this study was to assess the maturation of elasticity and colour of thin STSGs, compare deep dermal with muscular wound beds and to assess which scar scale is best suited to assess STSG maturation.

The decrease in all three overall scar scale scores indicates an improvement in STSG scar quality within 1 year after transplantation. Interestingly, this was not reflected in the subjective visual analogue scale, although it should be noted that the items pain, tension and itch measure different qualities. Three items on the Vancouver and Hamilton scar scales, pigmentation, vascularity and height/raising, assess similar qualities. However, the fourth item, pliability and irregularity, assesses different qualities, with ‘pliability’ in particular representing an important measure of scar quality and maturation. The items of the Manchester Scar Scale differentiate it from the other two with the qualities shine and colour. The item colour sums up vascularity and pigmentation whereas the Vancouver Scar Scale and the Hamilton Scar Scale both contain separate items to assess vascularity and pigmentation, and vascularity was assessed in the most detail with the Hamilton Scar Scale. Since vascularity is a major indicator of scar maturation, this item should be part of a comprehensive scar scale. The judgement of vascularity and pigmentation, however, is not easy to separate; therefore, Baryza et al. suggested the compression of the scar with plexiglass to facilitate the division of these two parameters [17]. Changes of contour and height were, however, assessed in the most detail with the Manchester Scar Scale. None of the scar scale parameters were sufficiently detailed to report the maturation of pigmentation. The maximum value, as well as the rating instructions of the items differ from scale to scale, which impedes comparison of values of equivalent items.

The analysed Cutometer parameters R0 and R3 increased significantly within the follow‐up period, indicating that the skin pliability of the STSG scars increased. The analysed parameters have a high interrater reliability and can be effectively used in the comparison of STSG scars and normal skin [12, 16]. Especially R0 is able to characterise skin stiffness [18]. However, 12 months postoperatively, the scars were still less pliable in comparison to the control. After a mean follow‐up time of 6 years, R0 was still reduced in STSG scars compared to normal skin by approximately 0.25 mm in a study by Kern et al. [19]. In the current study, R0 was 0.35 mm lower in STSG compared to normal skin after 12 months, which indicates that the STSG maturation process was still ongoing at the end of the follow‐up period. However, the pliability item of the Vancouver Scar Scale was not able to document this change of scar pliability. The scale of the item might not be precise enough to capture the STSG scar maturation process in this regard. This was assumed earlier by Rennekampf et al., who found no correlation between the pliability item of the Vancouver Scar Scale and Cutometer parameters in STSG donor sites [18]. The objective evaluation of STSG scar elasticity and pliability with a suction device, such as the Cutometer, is therefore superior to the Vancouver Scar Scale assessment, although the limited portability and acquisition costs of the device should be taken into account [20]. While the gross elasticity parameter R2 was consistent and similar to the control, the parameter R5, which represents the net elasticity, was initially higher than the control values. The higher the dimensionless parameters R2 and R5 values are, the more elastic the skin [21]. This means, in summary, the STSG scars were initially less pliable and more elastic than normal skin, although both values approach those of healthy skin over time.

While the assessment of scar colour appears to be simple, it should be considered that it is difficult to separate the assessment of the two most important factors determining skin colour: vascularisation and pigmentation [22]. These factors can be assessed reliably with the Mexameter [23]. The vascularity or erythema of the scar is an important indicator of scar maturation, and erythema is common in immature scars [8, 22]. The Erythema decreased continuously during the follow‐up but was still increased after 12 months in comparison to the control area. Both the Vancouver Scar Scale and the Hamilton Scar Scale show a significant continuous reduction of erythema and mirror the objective Mexameter measurements. This indicates that the Vancouver scar scale and Hamilton Scar Scale can be applied reliably to substitute the objective measurement, as Seo et al. suggested before for the Vancouver Scar Scale [20].

The results of the melanin index measurement reveal that most STSG scars were hypopigmented within the first 6 months after grafting. It is notable that the mean matched the control value after 12 months, which corresponds to Fitzpatrick score II–III [24]. The Vancouver Scar Scale only assesses if there is hyperpigmentation, hypopigmentation or normal pigmentation; however, the Hamilton Scar Scale further splits hyperpigmentation into 3 degrees but sums up normal pigmentation and hypopigmentation. This explains why the item pigmentation was more or less constant on both scar scales during the follow‐up period. The objective spectral absorption measurement, for example with the Mexameter, is therefore much more suitable to assess pigmentation and melanin of STSG scars within the first 12 months. Draaijers et al. also found that spectrophotometric analysis of scar vascularity and pigmentation is more reliable than scar rating by observation [25].

The comparison between the maturation of the centre and the margin of the scar revealed only minor differences. Except for the parameter R0 at 3 months postoperatively, the Cutometer values of the parameters R0, R2, R3 and R5 were not different in the centre and the margin of the scar, similar to the report of Kern et al. concerning the parameters R0, R2 and R8 [19]. The erythema index values also did not show significant differences depending on the location within the scar. However, the melanin index reached the baseline faster at the margin compared to the centre of the scar. This difference might be related to melanocytes migrating from the adjacent healthy skin into the STSG scar. Chadwick et al. proved histologically in the Duroc pig that melanocytes migrate from the adjacent skin into scar tissue, and therefore appeared in the centre of the scar with a time delay [26].

The comparison of deep dermal and muscular STSG showed that the overall scar scale scores were independent of the STSG wound bed, but individual items revealed differences. The lower pliability scores of deep dermal STSG on the Vancouver Scar Scale mean that the STSG scars were more pliable on deep dermal wound beds. The increased pliability coincided with the patients reporting a reduced feeling of tension. The objective assessment of elasticity with the Cutometer also revealed increased pliability of STSG on deep dermal wound beds compared to muscular wound beds. This difference can probably be attributed to the preserved subcutaneous tissue in the former, which is likely to allow more skin motion and decrease the skins' ability to return to its original state after deformation than the muscular wound bed. Likewise, when comparing STSG in pigs on subcutaneous tissue with grafts on muscle fascia, Rose et al. found less contraction and increased scar pliability, and furthermore found these differences to be larger in thin STSG [27]. In another study, the range of motion in extremity STSG was higher when grafted on fat compared to the muscular wound bed, which indicates increased pliability of STSG scars when grafted on fat also in humans [28]. Concerning skin colour, the objective evaluation with the Mexameter showed that there was less erythema in the muscular STSG group, which was in line with the differences in the item vascularity of the Hamilton Scar Scale. Furthermore, the melanin index was initially lower. Whereas Rose et al. found increased erythema and melanin content of STSG on muscle fascia in pigs [27]. However, it is important to note that Rose et al. compared STSG on subcutaneous tissue and our current study compared STSG on deep dermal grafts. Taken together, the findings suggest that erythema and melanin content are highest in STSG on deep dermal wound beds, followed by muscular/epifascial wound beds and lowest on subcutaneous wound beds. The findings suggest that deep dermis should be preserved, if possible, not only to maintain the thickness of the covering tissue layer, but also because STSG scar quality is superior on a deep dermal graft bed.

The study is limited by the lack of randomisation of patients. Therefore, patients with a muscular wound bed had sustained more severe and deeper‐reaching trauma than those with a deep dermal wound bed, which might have negatively influenced wound healing and scarring in this group. However, based on the study results, randomising patients with superficial skin defects to grafting on either a muscular or a deep dermal wound bed would not be ethically justifiable. To date, no clear scientific evidence exists on how defect size influences STSG outcomes. While STSG healing time in leg ulcers does not appear to be size‐dependent [29] large skin defects, such as extensive burn injuries, can trigger systemic impairment of microcirculation and a systemic immune response [30], which may adversely affect STSG healing and graft take rate. In our study, the size of the tissue defects, and thus the size of the STSGs, was larger in the muscular wound bed group. Although no patients with burn shock or with defect sizes greater than 10% TBSA were included, a potential influence of differing defect sizes between the two groups cannot be excluded. Furthermore, scars from different body locations were included, with a higher proportion of scars located on the trunk in the muscular group. The body location of the scar may influence the development of scar contracture. Furthermore, the study only included thin STSGs with a thickness of 2/10 mm and a mesh ratio of 1:1.5. This limits the generalisability of the study results to thicker grafts and different mesh ratios, as these STSG characteristics influence graft healing and maturation. For example, thin grafts show faster epithelialisation compared to thicker grafts [31], and unmeshed grafts generally yield superior aesthetic outcomes compared to meshed skin grafts [32]. However, this was a deliberate decision to reduce confounding by avoiding the inclusion of multiple STSG thicknesses and mesh ratios. Future studies could be expanded to include a range of graft thicknesses and mesh ratios; however, these should be analysed separately and would require a substantially larger study population. Larger multicentre studies should be conducted to address the limitations of this study with more homogeneous groups. Furthermore, future studies should include an additional group with STSGs on subcutaneous tissue, considering the findings of Rose et al., who reported better scar quality of STSGs on subcutaneous tissue compared to muscle fascia in pigs [27].

The 12‐month follow‐up revealed continuous maturation of the STSG scars; pliability increased and elasticity decreased, both approaching the norm values, melanin content increased and erythema decreased. However, only elasticity and the melanin content reached the level of healthy skin. The melanin content increased faster at the margin than at the centre of the scar. STSG on deep dermal wound beds are characterised by increased scar pliability and decreased elasticity when compared to muscular wound beds, however, erythema and melanin content were also increased.

## Funding

This work was supported by the Open Access Publishing Fund of the University of Tübingen.

## Ethics Statement

The study was approved by the local Research Ethics Committee (project number 289/2020BO2) in accordance with the Declaration of Helsinki.

## Consent

Oral and written informed consent was obtained from all participants.

## Conflicts of Interest

The authors declare no conflicts of interest.

## Data Availability

Research data are not shared.
